# Liposomal prednisolone inhibits vascular inflammation and enhances venous outward remodeling in a murine arteriovenous fistula model

**DOI:** 10.1038/srep30439

**Published:** 2016-07-27

**Authors:** ChunYu Wong, Taisiya Bezhaeva, Tonia C. Rothuizen, Josbert M. Metselaar, Margreet R. de Vries, Floris P. R. Verbeek, Alexander L. Vahrmeijer, Anouk Wezel, Anton-Jan van Zonneveld, Ton J. Rabelink, Paul H. A. Quax, Joris I. Rotmans

**Affiliations:** 1 Department of Nephrology, Leiden University Medical Center, Leiden, The Netherlands; 2 Einthoven Laboratory for Experimental Vascular Medicine, Leiden Medical Center, Leiden, The Netherlands; 3 Targeted Therapeutics, MIRA Institute for Biomedical Technology and Technical Medicine, University of Twente, The Netherlands; 4 Enceladus Pharmaceuticals BV, The Netherlands; 5 Department of Surgery, Leiden University Medical Center, Leiden, The Netherlands; 6 Leiden Academic Center for Drug Research, Leiden, The Netherlands

## Abstract

Arteriovenous fistulas (AVF) for hemodialysis access have a 1-year primary patency rate of only 60%, mainly as a result of maturation failure that is caused by insufficient outward remodeling and intimal hyperplasia. The exact pathophysiology remains unknown, but the inflammatory vascular response is thought to play an important role. In the present study we demonstrate that targeted liposomal delivery of prednisolone increases outward remodeling of the AVF in a murine model. Liposomes accumulate in the post-anastomotic area of the venous outflow tract in which the vascular pathology is most prominent in failed AVFs. On a histological level, we observed a reduction of lymphocytes and granulocytes in the vascular wall. In addition, a strong anti-inflammatory effect of liposomal prednisolone on macrophages was demonstrated *in vitro*. Therefore, treatment with liposomal prednisolone might be a valuable strategy to improve AVF maturation.

Arteriovenous fistulas (AVFs) are the preferred type of permanent vascular access for hemodialysis in view of their superior patency and lower rate of infectious complications, when compared to prosthetic arteriovenous grafts. However, the durability of AVFs is far from optimal with a 1-year primary patency of only 60%^1^. AVF maturation refers to the process of enlargement of the lumen of the access conduit and the concomitant increase in blood flow, both needed to allow safe cannulation and adequate hemodialysis treatment. The exact mechanisms underlying maturation failure are unknown, but impaired outward remodeling as well as intimal hyperplasia (IH) are both considered to contribute^2^. Animal studies have shown that the adaptive response that occurs upon AVF creation, is characterized by marked vascular inflammation as illustrated by the infiltration of macrophages and lymphocytes^3^,^4^ as well as the up regulation of pro-inflammatory cytokines^5^. In addition, recent clinical studies suggest that this inflammatory response is harmful for AVF maturation, by showing that plasma levels of C-reactive protein are inversely correlated with successful AVF maturation^6^.

Glucocorticoids (GCs) are well-known anti-inflammatory drugs that are widely used for the treatment of numerous inflammatory diseases^7^. Despite their excellent anti-inflammatory efficacy, the therapeutic use of systemic GCs is hampered by the high risk of occurrence of adverse side effects. Nanoparticle therapeutics such as liposomes have shown to facilitate selective delivery of drugs to inflamed tissues with a highly permeable microvasculature^8^, where liposomes are being phagocytized by macrophages^9^.

In the present study, we evaluated the feasibility and efficacy of liposomal prednisolone (L-Pred) to target the inflamed peri-anastomotic region of murine AVFs and assessed its effect on the morphometry and composition of the venous outflow tract. Subsequently, we assessed the efficacy of L-Pred to reduce the inflammatory profile of cultured macrophages and evaluated its effect on vascular smooth muscle cell (VSMC) proliferation *in vitro*.

## Results

### Surgical outcome

**Figure 1:**
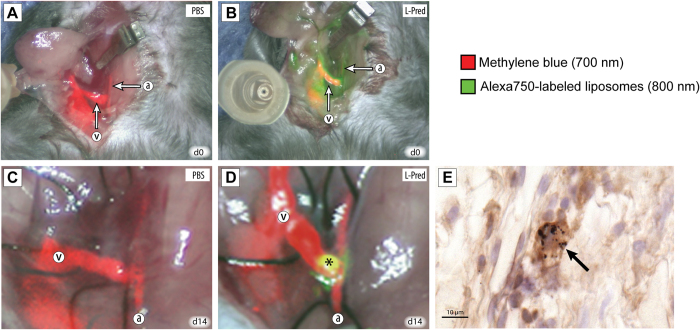
Localization of liposomes using near infrared fluoroscopy and immunohistochemistry. (**A,B**) *In vivo* imaging using NIRF of the AVF directly after creation (day 0) in mice that were injected with PBS (**A**) or L-Pred. (**B**) Red color overlay corresponds to the intravenously administered methylene blue visualized on the 700 nm channel. Green color overlay corresponds to the intravenously administered liposomes that are labeled with the Alexa-750 fluorochrome visualized on the 800 nm channel. (**C,D**) *In vivo* imaging using NIRF of the AVF at time of sacrifice (day 14) in mice that were injected with either PBS (**C**) or L-Pred (**D**) at day 0, 2, 5 and 10. (*) Extravasation of liposomes in the anastomotic area of the AVF. (**E**) Double staining against F4/80 and gold particles showing accumulation of gold-labeled liposomes in macrophages in the venous outflow tract (black arrows). (a) Artery, (v) Vein. Bar = 10 μm.

In total, 54 mice received an AVF (PBS; n = 10, L-PBS; n = 8 Pred; n = 9, L-Pred; n = 10, gold-containing PEG-liposomes; n = 3), of which 14 mice (26%) did not survive the surgical procedure. The number of deaths did not differ significantly between the different intervention groups when compared to the PBS treated group. In 6 of the 14 mice (43%), the cause of death was clearly due to postoperative bleeding in the surgical area. In the rest of the cases, the cause of death was unclear. The surviving mice showed normal behavior after recovering from the surgery until time to sacrifice. Figure 1, A-B and videos 1–2 illustrate the AVF directly after surgery as well as circulating liposomes shortly after intravenous administration.

With regard to adverse effects of systemic prednisolone phosphate therapy, animals that received either liposomal prednisolone phosphate or free prednisolone phosphate both showed disturbed wound healing in the ears that was caused by the placement of earmarks at the start of the surgical procedure. However, we did not observe any disturbance in the wound healing of the skin incisions located in the neck and upper leg. Apart from this adverse effect, the general condition of all the animals remained normal during the study.

### Patency

Using near infrared fluorescence (NIRF) (Fig. 1C,D and videos 3–4), we observed a 100% patency in all the groups except for the mice treated with prednisolone in which 2 out of the 9 (22%) AVFs were occluded at time of sacrifice. This difference in patency was not significant when compared to PBS (P = 0.15). These two occluded AVFs were excluded from further analysis.

### Distribution of liposomes in murine AVF

At 14 days after surgery, liposomes accumulated in the peri-anastomotic area of the AVF as assessed, by NIRF imaging (Fig. 1D and video 4). At this time, 4 days after the last injection, no circulating liposomes were detected. Histological analysis of the venous outflow tract in mice that were injected with the gold-labeled liposomes, showed marked accumulation of gold in F4/80(+) macrophages (Fig. 1E).

### Morphometric analysis

**Figure 2:**
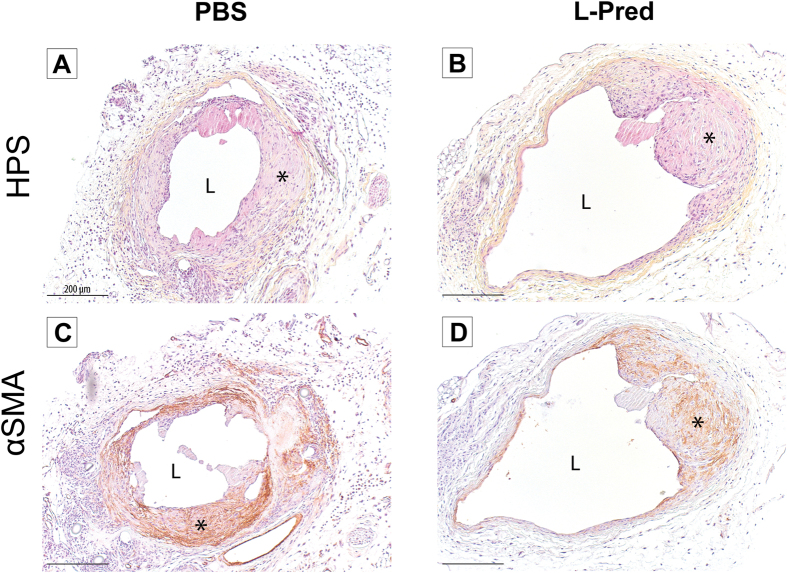
Histological stainings of the venous outflow tract of the AVF at day 14 after surgery. (**A,B**) Hematoxylin, phloxin and saffron staining (HPS). (**C,D**) Immunohistological staining against α-smooth muscle actin. (*) Intimal hyperplasia, (L) Lumen, Original magnification 100x. Bar = 200 μm.

**Figure 3:**
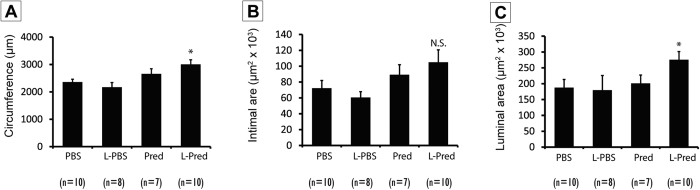
Histomorphometric parameters 14 days after AVF surgery. (**A**) Venous circumference. (**B**) Intimal area at the venous outflow tract. (**C**) Luminal area at the venous outflow tract. (*)*P* < 0.05 compared to PBS, (N.S) not significant compared to PBS.

Representative sections from the venous outflow tract of the AVF are shown in Fig. 2A,B. At day 14 after surgery, the mice that were treated with L-Pred showed a 27% larger circumference of the external jugular vein when compared to the PBS-treated group (P = 0.004), whereas no significant differences were observed in the Pred-treated mice (P = 0.195) or L-PBS treated mice (P = 0.396) (Fig. 3A).

No significant differences were observed in intimal area in the experimental groups (L-Pred: P = 0.052, Pred: P = 0.350, L-PBS: P = 0.494) when compared to PBS-treated mice (Fig. 3B). Immunohistochemical staining revealed that the vast majority of the cells in the intima stained positive for α-SMA in all the groups (Fig. 2C,D).

The luminal area in the L-pred treated mice showed a 47% increase compared to the PBS treated mice (P = 0.042), whereas the mice treated with Pred (P = 0.766) or L-PBS (P = 0.861) did not show a significant difference when compared to the PBS-treated mice (Fig. 3C).

### Inflammatory response in the AVF

**Figure 4:**
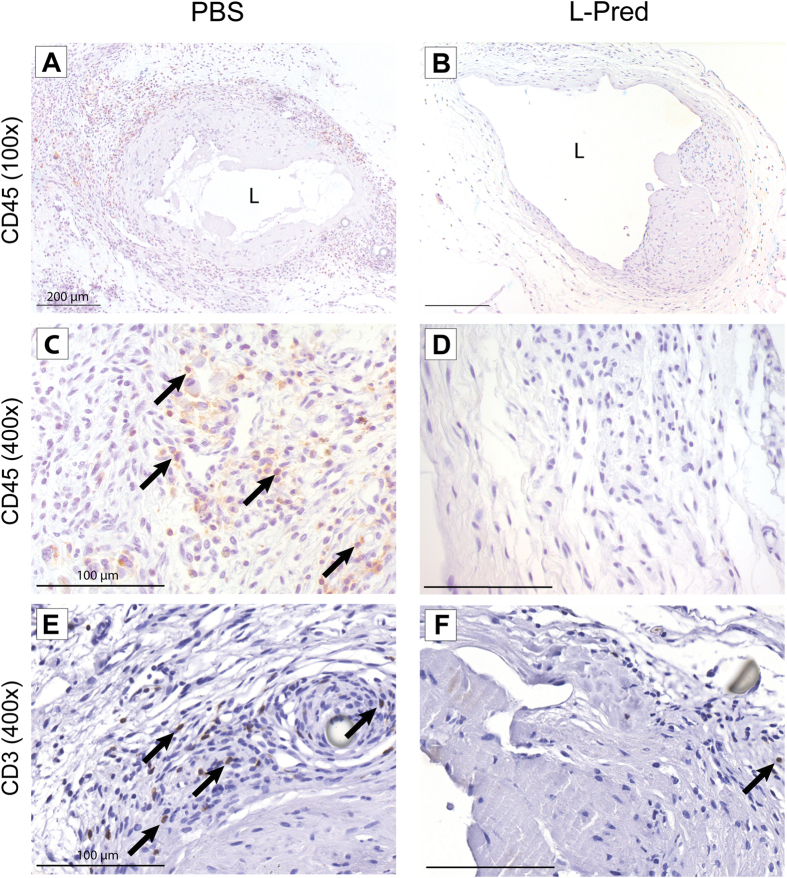
Representative sections of immunohistochemical stainings against CD45 and CD3 at 14 days after AVF surgery. (**A–D**) CD45(+) cells (black arrow) in the venous outflow tract of the AVF. (**E,F**) CD3(+) cells (black arrow) in the venous outflow tract of AVF. Original magnification (**A,B**): 100x. Bar = 200 μm, (**C–F**): 400x, Bar = 100 μm (L) Lumen.

**Figure 5:**
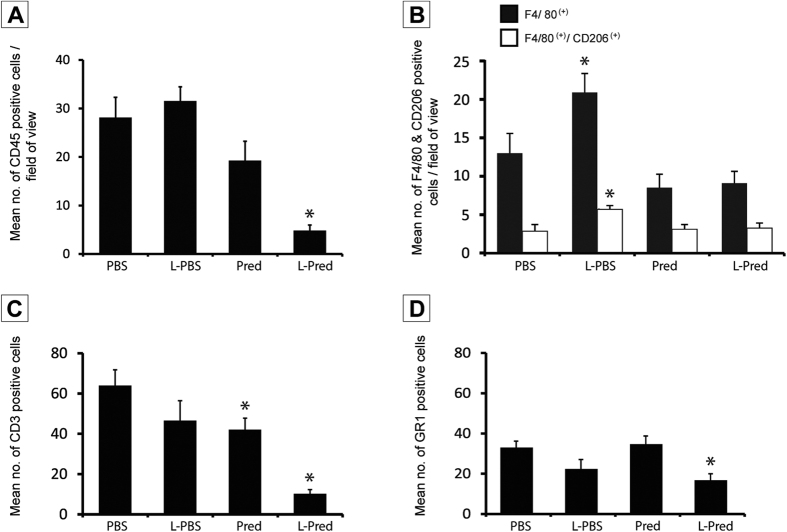
Quantification of immunohistological staining against different inflammatory cells at day 14. (**A**) CD45(+) leucocytes. (**B**) F4/80(+) macrophages and F4/80(+)CD206(+) M2 macrophages. (**C**) CD3(+) granulocytes. (**D**) GR1(+) granulocytes in the venous outflow tract of AVF at day 14. (*)*P* < 0.05 compared to PBS.

Representative sections of the immunohistochemical staining against CD45 and CD3 are shown in Fig. 4A–F. Immunohistochemical analysis of sections obtained from the venous outflow tract revealed that the mean number of CD45(+) cells present in the venous outflow tract of the L-Pred treated mice was reduced by 83%, when compared to the mice treated with PBS (P < 0.001). A trend towards reduction of CD45-positive cells in the venous vascular wall was observed in the Pred-treated mice, when compared to PBS (P = 0.069) (Fig. 5A). Additional immunohistochemical stainings for subsets of inflammatory cells revealed a 86% decrease in CD3(+) T-cells (p < 0.001) and a 51% decrease in GR1(+) granulocytes (P = 0.008) in the L-Pred treated group when compared to the PBS treated group (Fig. 5B,D).

### Inflammatory effect of L-Pred in cultured macrophages

**Figure 6:**
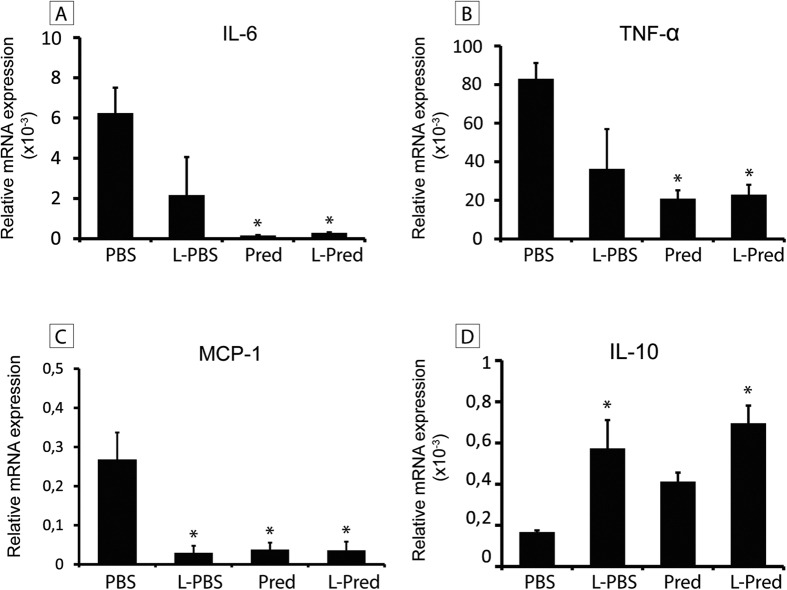
mRNA expression levels of pro- and anti-inflammatory cytokines in macrophages *in vitro* after incubation with PBS (control medium), L-PBS, Pred and L-Pred. (**A–C**) Relative mRNA expression of pro-inflammatory cytokines (IL-6, TNF-α and MCP-1) in M1 macrophages and (**D**) the anti-inflammatory cytokine IL-10 in M2 macrophages. The relative expression is normalized against GAPDH. (*)*P* < 0.05 compared to PBS.

To study the inflammatory effects on a mRNA level, we exposed murine M1 and M2 macrophages to L-Pred, L-PBS, Pred and PBS. Upon incubation with L-Pred and Pred, a strong reduction was observed in the expression of pro-inflammatory genes including TNF-α, MCP-1 and IL-6 (all P < 0.01) by M1 polarized macrophages, whereas M2 macrophages showed a significant increase in the expression of the anti-inflammatory cytokine IL-10 after addition of L-Pred and L-PBS (both P < 0.01) when compared to the incubation with PBS (Fig. 6A–D).

### Effect of liposomal prednisolone on the expression of MMPs and TIMP.

**Figure 7:**
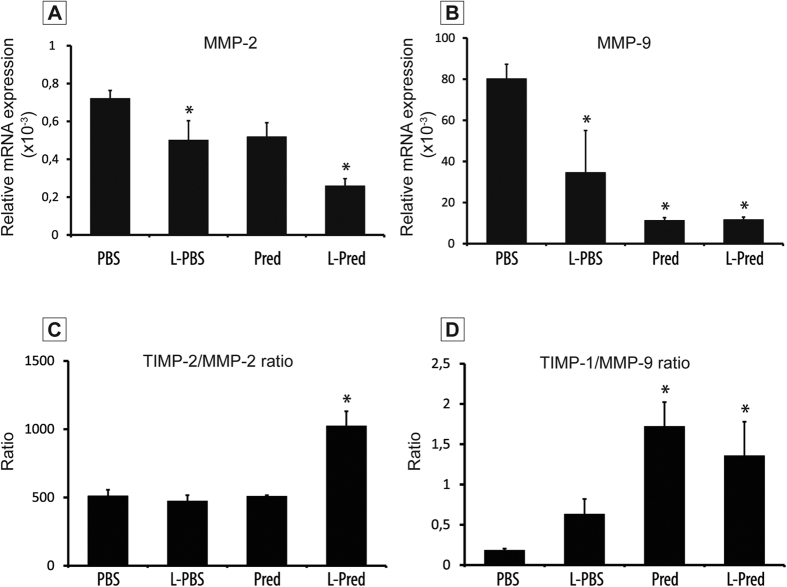
mRNA expression levels of MMP-2, MMP-9 and the ratios of TIMP-2/MMP-2 and TIMP-1/MMP-9 in macrophages after incubation with PBS (control medium), L-PBS, Pred and L-Pred. (**A,B**) Relative mRNA expression of MMP-2 and MMP-9. (**C,D**) Ratios of TIMP-2/MMP-2 and TIMP-1/MMP-9. Expression was normalized against GAPDH. (*)*P* < 0.05 compared to PBS.

We measured the expression of matrix metalloproteinases (MMP-2 and MMP-9) and their endogenous inhibitors (TIMP-2 and TIMP-1, respectively) on RNA level in M1 macrophages. Upon incubation with L-Pred, both MMP-2 and MMP-9 expression decreased when compared to PBS incubation. In addition, both the TIMP1/MMP-9 and the TIMP2/ MMP-2 ratio increased upon incubation with L-Pred when compared to PBS incubation (Fig. 7A–D).

## Discussion

In the present study, we demonstrate that intravenously administered liposomes selectively target the anastomotic region and can effectively improve venous outward remodeling of murine AVF when prednisolone is encapsulated in the liposomes.

Liposomes are one the most prominent drug carrier systems currently available, due to their relative easy preparation using biocompatible components and the high drug payload^10^. Moreover, liposomes can hold their payload while circulating in the vasculature until they extravasate and accumulate at sites of inflammation as a result of enhanced vascular permeability in inflamed tissues, thereby reducing possible systemic adverse side effects^11^. The PEG-coating of liposomes is designed to improve their bioavailability in inflamed tissues as it serves as a steric barrier that minimizes liposomal uptake by circulating mononuclear cells^12^. Once extravasated, the liposomes are recognized as foreign particles and phagocytized by macrophages^10^. Using NIRF and immunohistochemistry, we demonstrated that liposomes indeed accumulate in macrophages in the anastomotic area of murine AVF. These results are consistent with previous studies in rabbits^13^ and humans^14^ which revealed marked accumulation of liposomes in macrophages within inflammatory atherosclerotic plaques.

GCs are powerful anti-inflammatory drugs that act through binding to cytosolic glucocorticoid receptors in target cells, leading to a reduction in the expression of pro-inflammatory cytokines and diminished recruitment of inflammatory cells^15^,^16^. Despite these potent anti-inflammatory effects, chronic and systemic therapeutic use of GCs is limited by the high incidence of serious adverse effects^16^. Moreover, as a result of rapid clearance from the circulation, systemic administration of GCs results in low efficacy of drug delivery at the target location. Therefore, encapsulation of GCs in liposomal nanoparticles has great potential to enhance the therapeutic effect of GCs in inflamed tissues. Previous studies on L-Pred in murine model of arthritis^17^ have underscored this potential, as complete remission of the inflammatory response was shown upon a single injection, which was superior to the effect of unencapsulated prednisolone.

Preclinical studies have shown that GCs reduce the formation of both arterial and venous stenotic lesions^18^,^19^,^20^. However, the role of inflammation in vascular remodeling upon AVF surgery has not been elucidated yet. Although various animal studies^3^,^4^,^5^,^21^ revealed infiltration of inflammatory cells in the venous outflow tract in the early phase after AVF surgery, it is unclear whether this inflammatory response directly contributes to maturation failure. Our experiments revealed that L-Pred resulted in inhibition of vascular inflammation in the AVF, as illustrated by a significant reduction of lymphocytes and granulocytes in the venous outflow tract. Interestingly, treatment with unencapsulated prednisolone resulted solely in a modest reduction in inflammatory cells, suggesting that liposomal encapsulation indeed resulted in a higher concentration of prednisolone in the venous outflow tract. As shown *in vitro,* L-Pred induced a conversion towards an anti-inflammatory profile of macrophages, as demonstrated previously^22^. Moreover, liposomes themselves contribute to the reduction of the pro-inflammatory profile of macrophages, as illustrated by the reduction of pro-inflammatory cytokines in M1 macrophages upon stimulation with L-PBS. This shift towards an anti-inflammatory profile of macrophages might have inhibited the recruitment of lymphocytes and granulocytes to the injured vessels of the AVF. Alternatively, prednisolone might have had a direct effect on lymphocytes and granulocytes as GCs can easily pass the cellular membrane of macrophages in both directions.

While previous studies evaluated the therapeutic effect of GCs to inhibit IH in various vascular injury models^18^,^19^,^20^, none of these studies focused on its effect on vascular remodeling. The question arises how treatment with L-Pred resulted in enhanced outward remodeling in the venous outflow tract of AVF. In order to investigate whether L-Pred influenced VSMC proliferation and collagen production, we directly stimulated cultured murine VSMCs with L-Pred. However, no significant effect on VSMC proliferation and collagen synthesis was observed. (data not shown).

MMPs are a group of proteolytic enzymes involved in vascular remodeling by facilitating the turnover of extracellular matrix components such as collagen and elastin^23^. The effect of MMPs on the vascular remodeling depend on the specific MMPs that are activated and the type of vascular injury^24^. Previous studies in a porcine balloon angioplasty model in peripheral arteries revealed that treatment with the MMP-inhibitor marimastatin resulted in reduced constrictive arterial remodeling in favor of expansive remodeling^25^. The exact contribution of MMPs in vascular remodeling in AVF stills remains to be elucidated. Previous studies suggest that the effect of MMP activation on vascular remodeling in AVF might differ between the arterial and venous segment. Indeed, Castier *et al.* observed that enhanced MMP-9 activity coincided with increased outward remodeling in the arterial segment of murine AVF, whereas studies by Nieves Torres *et al.*^26^ revealed that MMP inhibition enhanced venous outward remodeling in AVF. In the latter study, adventitial delivery of a small hairpin RNA against the MMP ADAMTS-1, resulted in reduced macrophage infiltration, decreased MMP-9 activity and enhanced venous outward remodeling in murine AVF. Although we have not been able to quantify MMP activity *in vivo*, we speculate that the enhanced venous outward remodeling in murine AVF that occurred upon treatment with L-Pred is mediated by the dampened inflammatory response and decreased MMP activity in macrophages. In addition, a recent clinical trial evaluating the efficacy of the MMP-inhibitor doxycycline to inhibit growth of abdominal aortic aneurysms resulted in an unexpected acceleration of the aneurysmal growth^27^. These data suggest that in certain vascular disease conditions, inhibition of MMPs could enhance outward remodeling. Interestingly, elegant studies by Nieves Torres and coworkers^26^ suggest a similar effect of MMP inhibiton on venous outward remodeling in AVF. Indeed, adventitial delivery of a small hairpin RNA against the MMP ADAMTS-1, resulted in reduced macrophage infiltration, decreased MMP-9 activity and enhanced outward remodeling in murine AVF. Although we have not been able to quantify MMP activity *in vivo*, we speculate that the enhanced outward remodeling in murine AVF that occurred upon treatment with L-Pred is mediated by the dampened inflammatory response and decreased MMP activity in macrophages.

In contrast to its effect on outward remodeling, no inhibitory effect of L-Pred on IH in the venous outflow tract was observed. These results deviate from other preclinical studies that evaluated the therapeutic effect of GCs in other vascular injury models, that have reported a strong inhibitory effect of dexamethasone on IH^18^,^19^,^20^. This discrepancy may result from a difference in potency between prednisolone and dexamethasone to inhibit VSMC proliferation^28^. Alternatively, it may relate to the difference in pathophysiological stimuli that contribute to IH after arterial injury, when compared to venous IH in AVF. While hemodynamic stimuli are considered to be of vital importance for IH^29^,^30^, the contribution of inflammation to IH in AVF might be limited, as suggested by our results. Interestingly, our study showed an increase in OR that was accompanied by a trend towards an increase in IH, which could potentially reduce the luminal area and therefore blood flow. Although the exact explanation for this phenomenon is not fully elucidated, we believe that this is due to the fact that both OR and IH are processes that involves VSMC proliferation. As a consequence, interventions that facilitate OR might therefore also result in a (modest) stimulation of IH. Of note, a stimulatory effect of an intervention on OR is more important for the ultimate luminal surface area than the coinciding effect on IH, as there is a quadratic relationship between radius and surface area of the vessel.

Preclinical studies in pigs^4^ revealed that the inflammatory response in the venous outflow tract of AVF is temporal, peaking in the early weeks after surgery. This acute, localized inflammatory response makes the application of L-Pred particularly appealing as a short-term treatment in the first weeks after surgery.

In conclusion, liposomal prednisolone reduces the local inflammatory response and stimulates venous outward remodeling in murine AVF. Therefore, treatment with liposomal prednisolone might be valuable strategy to reduce AVF non-maturation. The efficacy of liposomal prednisolone to enhance AVF maturation in ESRD patients will be evaluated in the LIPMAT trial (clinicaltrial.gov ID NCT0249566), a double-blind, randomized, placebo-controlled trial that will commence in Q4 of 2015.

## Methods

### Liposomes

Prednisolone polyethylene glycol-coated (PEG)-liposomes were prepared as described in the supplemental data and contained 2 mg prednisolone phosphate per ml. The PEG-liposomes were labeled with Alexa 750 succinimidyl (Invitrogen, Carlsbad, CA, USA) and contained either prednisolone phosphate (Fagron, Capelle aan den IJssel, The Netherlands) or PBS. In order to trace the liposomes microscopically, additional PEG liposomes were coupled to gold particles (Nanoprobes, Yaphank, NY, USA).

### Animal experiment

The Institutional Committee for Animal Welfare at the Leiden University Medical Center approved all animal experiments that were performed in accordance with the relevant guidelines and regulations. Adult male C57bl6 mice aged 10–11 weeks were used for the experiments and received an unilateral AVF between the dorsomedial branch of the external jugular vein and the common carotid artery in an end-to-side manner as previously described^3^ (supplemental data). At the end of the procedure, either L-Pred (10 mg/kg bodyweight), Pred (10 mg/kg bodyweight) (Fagron), L-PBS or PBS was injected intravenously. The dose was determined according to the guidelines of the Food and Drugs Administration, in which the safe starting dose level of prednisolone was set at 1 mg/kg. The injection volume of L-PBS and PBS was equal to the injected volume of the L-Pred and Pred (200 μL). Due to a positive additive effect of multiple injections as compared to a single injection^31^, we injected the mice intravenously at day 2,5 and 10. In addition, 3 separate mice received a single bolus injection intravenously of gold-labeled empty liposomes (130 μL) at day 2 to evaluate liposomal accumulation in the AVF microscopically. All mice were sacrificed 14 days after the surgical procedure.

### Near-infrared fluorescence imaging

To assess the accumulation of the liposomes macroscopically, we used the near infrared fluorescence (NIRF) imaging technique^3^ directly after surgery and at time of sacrifice. After positioning of the Fluorescence Assisted Resection and Exploration (FLARE) imaging system (Center for Molecular Imaging, Boston, MA, USA) approximately 46 cm above the AVF, 200 μl 1% methylene blue (Sterop, Belgium) dissolved in saline was injected in the left femoral vein to assess the patency of the AVF. Video images were captured with 30-frames-per-second. Multiple video channels (color video, 700 nm and 800 nm) were obtained simultaneously with 60 milliseconds exposure times. After computer-controlled image acquisition, color video and NIRF images were displayed individually and merged in real-time. The 800 nm and 700 nm channel were used for imaging the liposomes and methylene blue, respectively.

### Tissue harvesting and processing

Fourteen days after surgery, the mice were anesthetized (supplemental data) whereupon the AVF was dissected and assessed using NIRF as described above. Next, the inferior vena cava was transsected followed by mild pressure perfusion fixation with 4% formalin through an intracardiac perfusion. The tissue was processed to paraffin and 5 μm-thick sections of the venous outflow tract were made perpendicular to the vein with an interval of 150 μm.

### Morphometric analysis

Morphometric analysis was performed on Weigert’s elastin stained sections using quantitative imaging software (Qwin, Leica, Wetzlar, Germany). The intimal area was calculated by subtracting the luminal area from the area within the internal elastic lamina (IEL). The circumference of the vessel was determined by measuring the length of the IEL. Results are expressed as mean ± SEM.

### Immunohistochemical staining and analysis

All immunohistochemical quantifications were performed on the first 3 venous sections starting from the anastomosis per AVF. Slides were digitized using an automated microscopic scanner (Panaoramic digital MIDI slice scanner, 3DHISTECH, Hungary). Detailed protocols of the immunohistochemical stainings are listed in supplemental data.

Serial sections from each AVF were stained with the following antibodies: anti- α-smooth muscle actin (α-SMA) for vascular smooth muscle cells and myofibroblasts (Dako, Glostrup, Denmark); anti-CD45 for leukocytes (BD Pharmingen, San Diego, California, USA); anti-F4/80 for macrophages (Abcam, Cambridge, UK); anti-CD-206 for anti-inflammatory (M2) macrophages (Abcam); anti-CD-3 for T-lymphocytes (Abcam) and GR-1 for granulocytes (from G. Kraal, VUMC, Amsterdam, The Netherlands). To distinguish anti-inflammatory (M2) macrophages from the whole macrophage population anti-F4/80 and anti-CD-206 antibodies were combined in a double immunofluorescence staining.

For the immunohistochemical analysis of the CD206-F4/80 and CD45 staining, the number of positive cells was counted in two random fields of view using a 400x magnification from which the mean was calculated. In view of the limited presence of CD3+ and GR1+ cells, quantification of these cells was performed by counting all positive cells that were present in the venous segment of the AVF. Gold-labeled liposomes were visualized using the LI Silver enhancement kit (Nanoprobes) and combined with the F4/80 staining.

### Generation and stimulation of bone marrow derived macrophages

To generate bone marrow-derived macrophages (BMDM) femurs of healthy C57Bl/6 male mice (n = 3) were flushed with sterile PBS. Total bone marrow progenitor cells (BMPCs) were centrifuged at 300 g at 4 °C for 10 min and incubated for 5 min on ice with erythrocyte lysis buffer (Sigma-Aldrich R7757, St. Louis, MO, USA). After erythrocytes were depleted, remaining cells were washed three times with PBS, centrifuged at 300 g at 4 °C for 10 min, and resuspended in RPMI medium supplemented with 20% fetal calf serum (FCS), 2 mmol/L l-glutamine, 100 U/ml penicillin, 100 μg/ml streptomycin (all from PAA, Colbe, Germany). To promote differentiation of BMPCs towards macrophages, cultured medium was supplemented with 10 ng/ml of macrophage colony-stimulating factor (M-CSF) (Peprotech, Rocky Hill, NJ, USA). Cells were seeded at a density of 1.0 * 10^6^ cells per well, and the medium was replaced on day 3 and 5.

On day 7, cells were stimulated either with LPS (100 ng/ml) and IFN-gamma (10 ng/ml) to differentiate them towards pro-inflammatory (M1) phenotype or with IL-4 (10 ng/ml) and IL-13 (10 ng/ml) (all from Preprotech) for anti-inflammatory (M2) phenotype. On the same day Pred (10 μg/ml), L-Pred (10 μg/ml), L-PBS or control medium was added to the cells (n = 3). After 24 hours the supernatant was collected and cells were lysed for RNA isolation.

### RNA isolation, cDNA synthesis and qPCR

Total RNA was extracted from the macrophages cells using Trizol reagent (Invitrogen, Carlsbad, CA, USA). RNA was reverse transcribed using a 5-minute 65 °C incubation of 1 μg total RNA with deoxyribonucleotide triphosphates (Invitrogen) and random primers (Invitrogen). c-DNA was synthesized using an M-MLV First-Strand Synthesis system (Invitrogen), and used for quantitative analysis of mouse genes (supplemental data) with an SYBR Green Master Mix (Applied Biosystems, Foster City, CA, USA). Levels of gene expression were determined by normalization to murine glyceraldehyde 3-phosphate dehydrogenase (GAPDH).

### Statistical analysis

All data except the patency outcome were expressed as mean ± SEM. SPSS 20.0 was used for all statistical calculations. Except for the data on AVF patency and animal survival, all measurements were analyzed statistically using One-way ANOVA with Dunett post-hoc test with the PBS group as the reference category. A Fisher’s exact test was used for the data on AVF patency and animal survival. A *P*-value < 0.05 was considered statistically significant.

## Additional Information

**How to cite this article**: Wong, C.Y. *et al.* Liposomal prednisolone inhibits vascular inflammation and enhances venous outward remodeling in a murine arteriovenous fistula model. *Sci. Rep.* **6**, 30439; doi: 10.1038/srep30439 (2016).

## Supplementary Material

Supplementary Video 1

Supplementary Video 2

Supplementary Video 3

Supplementary Video 4

Supplementary Information
