# Supplementary material for: Liposomal prednisolone inhibits vascular inflammation and enhances venous outward remodeling in a murine arteriovenous fistula model
Source: Sci Rep. 2016 Jul 27;6:30439. doi: 10.1038/srep30439 (PMC4962038; doi:10.1038/srep30439)
Supplement: Supplementary Information [file srep30439-s5.doc]

**Supplementary data**

**Liposomal prednisolone inhibits vascular inflammation and enhances venous outward remodeling in a murine arteriovenous fistula model**

ChunYu Wong^1,2^, Taisiya Bezhaeva^1,2^, Tonia C. Rothuizen^1,2^, Josbert M. Metselaar^3,4^, Margreet R. de Vries^2,5^, Floris P.R. Verbeek^5^, Alexander L. Vahrmeijer^5^, Anouk Wezel^5,6^, Anton-Jan van Zonneveld^1,2^, Ton. J. Rabelink^1,2^, Paul H.A. Quax^2,5^ and Joris I. Rotmans*^1,2^

^1^ Department of Nephrology, Leiden University Medical Center, Leiden, The Netherlands;

^2^ Einthoven Laboratory for Experimental Vascular Medicine, Leiden Medical Center, Leiden, The Netherlands;

^3^ Targeted Therapeutics, MIRA Institute for Biomedical Technology and Technical Medicine, University of Twente, The Netherlands

^4^ Enceladus Pharmaceuticals BV

^5^ Department of Surgery, Leiden University Medical Center, Leiden, The Netherlands

^6^ Leiden Academic Center for Drug Research, Leiden, The Netherlands.

**METHODS**

**Liposomal prednisolone preparation**

Prednisolone phosphate polyethylene glycol-coated (PEG)-liposomes were prepared by injecting 1 mL of an alcoholic lipid solution of 1 Molar (containing dipalmitoyl phosphatidyl choline and dipalmitoyl phosphatidyl glycerol, both from Lipoid GmbH, Germany, and cholesterol (Sigma Aldrich, saint Louis, MO, USA)) in a molar percentage of 62%, 5%, and 33% of total lipid content, respectively), in 9 mL of an aqueous solution of 100 mg/ml prednisolone phosphate disodium salt (Fagron, Capelle aan den IJssel,The Netherlands). Subsequently the 10 ml crude liposome dispersion was sized by multiple extrusion at 60 degrees C using a medium pressure extruder (Lipex) equipped with two stacked polycarbonate membrane filters with 100 nm pores. Alcohol and free prednisolone phosphate (not incorporated in liposomes) were removed by ultrafiltration and replacement of the filtrate with clean phosphate buffered 0.9% saline (pH 7.4). The resulting formulation consisted of liposomes of approximately 105 nm in diameter as measured by dynamic light scattering, with a polydispersity index of 0.05 and a zeta-potential of approximately -30 mV. Content determination was done by extraction using the organic phase for lipid determination (HPLC followed by evaporative light scattering detection) and the aqueous phase to assess the prednisolone phosphate content (UV spectrophotometry at 254 nm). The liposomes contained approximately 2 mg prednisolone phosphate/mL and 70 µmol total lipid/ml. “Empty” liposomes (L-PBS) were prepared in the same manner using phosphate buffered saline instead of the aqueous prednisolone phosphate solution.

The liposomes were labeled with Alexa 750 by first mixing PEG(2000)-DSPE-NH2 and PEG(2000)-DSPE (Avanti Polar Lipids, Birmingham, AL, USA) in a 1:1 molar ratio in 0.1 M sodium bicarbonate solution at pH 8.3. This mixture was then heated at 60°C and Alexa 750 succinimidyl (Invitrogen, Carlsbad, CA, USA) was added, which led to coupling of Alexa 750 to the NH2-PEGylated lipid. This mixture was subsequently added to the liposomes and mixed under repeated temperature cycling between 60°C and room temperature, allowing the PEGylated and Alexa-conjugated lipid to insert in the liposome bilayer. In order to trace the liposomes at a microscopic level, gold-containing PEG-liposomes were prepared by adding to the PEG-liposome lipid mixture described above 0.1mol% dipalmitoyl phosphatidyl ethanolamine coupled to 1.4 nm gold particles (Nanoprobes, Yaphank NY, USA). After formation of the crude liposome dispersion multiple extrusion was performed using the same method as with prednisolone phosphate PEG-liposomes and identical size characteristics were obtained (103 nm, polydispersity index 0.08). The final formulation contained 10 μmol lipid/ml.

**Surgical procedure**

The animal was anesthetized using isoflurane followed by shaving and disinfection of the skin in the ventral neck area and fixed in a supine position on a heating blanket. The mouse was then injected with buprenorphin (0.1 mg/kg) (MSD, Whitehouse Station, NJ, USA) and 0.5 mL saline. Under a dissecting microscope (Leica, Wetzlar, Germany), an incision in the ventral midline of the neck area was made, followed by a dissection of the right dorsomedial branch of the external jugular vein and ipsilateral common carotid artery after the excision of the sternocleidomastoid muscle using a heat cauterizer. Next, after applying a vascular clamp (S&T, Neuhausen, Switzerland) on the proximal and distal artery an approximate 1 mm incision was made using a microscissor (Fine Science Tools, Heidelberg, Germany) and the lumen was rinsed with a heparin solution (100 IU/ml) (LEO Pharma, Ballerup, LLDenmark). The vein was then clamped proximally and ligated distally, followed by a transection just proximal to the ligation. After rinsing the vein with a heparin solution, an end-to-side anastomosis was created using 10.0 interrupted sutures (BBraun, Melsungen, Germany). Halfway during the suturing procedure, heparin (0.2 IU/gram bodyweight) together with 200 µL of either L-Pred (10 mg/kg bodyweight), Pred (10 mg/kg bodyweight), L-PBS or PBS was injected intravenously. After completion of the anastomosis, the remaining clamps were removed and patency was assessed. The skin was closed with a 6.0 running suture (BBraun, Melsungen, Germany). Following completion of the surgery 0.5 mL of saline was injected subcutaneously and the mice were kept warm until recovery.

**Sacrification and tissue harvesting**

Upon sacrifice, mice received an intraperitoneal injection with an anesthetic-mixture containing midazolam (5 mg/kg) (Roche, Basel, Switzerland), medetomidine (0.5 mg/kg) (Orion, Espoo, Finland) and fentanyl (0.05 mg/kg) (Janssen, High Wycombe, UK) whereupon a reincision was made over the scar. The AVF was dissected and assessed using NIRF as described above. After a thoracotomy, the inferior vena cava was transsected followed by a mild pressure perfusion fixation with 4% formalin through an intracardiac perfusion.

**Immunohistochemistry and Immunofluorescence**

For immunohistochemical stainings, deparaffinization and hydration was followed by a treatment with 1% hydrogen peroxide and 5% bovine serum albumin in order to block the endogenous peroxidase and aspecific binding sites respectively. To unmask the antigens and epitopes antigen retrieval with Citrate Buffer (pH6) at 95-100 °C for 1 hour was done for CD-3, CD-206 and F4/80 antibodies. After 15 min blocking with 3% BSA (Sigma-Aldrich, , St. Louis, MO, USA) in PBS sections were incubated overnight at room temperature with specific antibodies to goat anti-mouse F4/80 (Abcam, Cambridge, UK), rabbit anti-mouse CD-206 (Abcam, Cambridge, UK), rabbit anti-mouse CD-3 (Abcam, Cambridge, UK) and goat anti-mouse GR-1 (from G. Kraal, VUMC, Amsterdam, The Netherlands). Control sections were incubated with rabbit IgG at 1:200 or 1:1000 dilution for CD-3 or CD-206 respectively or goat IgG at 1:100 or 1:300 dilution for F4/80 or GR-1 respectively. On the next day sections were briefly rinsed in 1% BSA (Sigma-aldrich) in PBS and incubated for 1 hour at room temperature with appropriate secondary antibodies that were goat anti-rabbit IgG labeled with Alexa-488 or goat anti-rat IgG labelled with Alexa-568 (Molecular Probes) for F4/80, CD-206 double immunofluorescence staining or horseradish peroxidase conjugated (Jackson ImmunoResearc bh,Westgrove, PA) goat anti-rat IgG at 1:200 dilution or peroxidase-based EnVision kit (DAKO, Glostrup, Denmark) for GR-1 or CD-3 respectively. For horseradish peroxidase-based stainings (CD-3 and GR-1) immunoreactive tissue was then developed by using a 3,3′-diaminobenzidine peroxidase substrate kit (Dako) and counterstained with hematoxylin. For immunofluorescence F4/80+/CD-206+ double staining sections were coverslipped with ProLong® Gold Antifade with DAPI (Life technologies, California, US) to counterstain nuclei. All slides were further digitized by an automated microscopic scanner (Panoramic digital MIDI slice scanner, 3DHISTECH, Hungary). All histologic evaluations were performed in a blinded manner on the first three venous sections upstream from anastomotic area. Venous part was chosen because most of the stenotic lesions in human AVFs occur in the venous outflow tract. For CD-3 and GR-1 immunohistochemistry the total number of positive cells per section was counted. F4/80 single or CD-206 double positive immunofluorescence staining were quantified as total number of positive cells per field of view in x400 magnification.

**Table 1**

**Primers used for *in vitro* experiments**

| **Gene** | **Forward primer** | | **Reversed primer** |
| --- | --- | --- | --- |
| IL-6 | CTGCAAGAGACTTCCATCCAG | | AGTGGTATAGACAGGTCTGTTGG |
| TNF | CCCTCACACTCAGATCATCTTCT | | GCTACGACGTGGGCTACAG |
| IL-10 | GCTGGACAACATACTGCTAACC | | CCCAAGTAACCCTTAAAGTCCTG |
| MCP-1 | GCACCAGCCAACTCTCAC | | CTTCTTGGGGTCAGCACAG |
| MMP2 | CCGAGGACTATGACCGGGATA | | GGGCACCTTCTGAATTTCCA |
| MMP9 | CTGGCGTGTGAGTTTCCAAAAT | | TGCACGGTTGAAGCAAAGAA |
| TIMP1 | ACACCCCAGTCATGGAAAGC | | CTTAGGCGGCCCGTGAT |
| TIMP2 | GTTTATCTACACGGCCCCCTCTT | | ATCTTGCCATCTCCTTCTGCCTT |
| GAPDH | ACTCCCACTCTTCCACCTTC | | CACCACCCTGTTGCTGTAG |
| |  | | --- | | |  | |
|  | |  | |

**SUPPLEMENTAL VIDEOS**

**Video 1-4**. In vivo NIRF imaging of the AVF at day 0 and day 14 in mice treated with intravenously administered PBS or Alexa-750 labeled L-Pred. The red color overlay corresponds to the intravenously administered methylene blue visualized on the 700 nm channel. Green color overlay corresponds to the intravenously administered liposomes that are labeled with the Alexa-750 fluorochrome visualized on the 800 nm channel. The animal was oriented in such a fashion that the top of the screen resembles the cranioventral side.

**Video 1.** In vivo NIRF imaging of the AVF directly after creation (day 0) in an animal that was injected with PBS. Using methylene blue, we confirmed the patency of the AVF.

**Video 2.** In vivo NIRF imaging of the AVF directly after creation (day 0) in an animal that was injected with L-Pred. Intravascular circulating liposomes was confirmed together with AVF patency.

**Video 3.** In vivo NIRF imaging of the AVF at time of sacrification in an animal that was injected intravenously with PBS at day 0,2,5 and 10. The neck was dissected bilaterally in order to expose the contralateral side. Suture threads were placed around the venous outflow tract, proximal and distal common carotid artery of the AVF and around the contralateral branch of the external jugular vein. AVF patency was confirmed after administration of intravascular methylene blue.

**Video 4.** In vivo NIRF imaging of the AVF at time of sacrification in an animal that was injected intravenously with L-Pred at day 0, 2, 5 and 10. Suture threads were placed around the venous outflow tract, proximal and distal common carotid artery of the AVF and around the contralateral branch of the external jugular vein. At day 14, liposomal extravasation was observed around the anastomosis of the AVF and in the wound edges. No circulating liposomes were observed.
